# FtsZ Reorganization Facilitates Deformation of Giant Vesicles in Microfluidic Traps**

**DOI:** 10.1002/anie.202001928

**Published:** 2020-09-17

**Authors:** Kristina A. Ganzinger, Adrián Merino‐Salomón, Daniela A. García‐Soriano, A. Nelson Butterfield, Thomas Litschel, Frank Siedler, Petra Schwille

**Affiliations:** ^1^ Department of Cellular and Molecular Biophysics Max Planck Institute of Biochemistry Am Klopferspitz 18 82152 Martinsried Germany; ^2^ Living Matter AMOLF P.O. Box 41883-1009 DB Amsterdam The Netherlands

**Keywords:** cell division, membranes, microfluidics, protocells, vesicles

## Abstract

The geometry of reaction compartments can affect the local outcome of interface‐restricted reactions. Giant unilamellar vesicles (GUVs) are commonly used to generate cell‐sized, membrane‐bound reaction compartments, which are, however, always spherical. Herein, we report the development of a microfluidic chip to trap and reversibly deform GUVs into cigar‐like shapes. When trapping and elongating GUVs that contain the primary protein of the bacterial Z ring, FtsZ, we find that membrane‐bound FtsZ filaments align preferentially with the short GUV axis. When GUVs are released from this confinement and membrane tension is relaxed, FtsZ reorganizes reversibly from filaments into dynamic rings that stabilize membrane protrusions; a process that allows reversible GUV deformation. We conclude that microfluidic traps are useful for manipulating both geometry and tension of GUVs, and for investigating how both affect the outcome of spatially‐sensitive reactions inside them, such as that of protein self‐organization.

## Graphical abstract

Microfluidic vesicle traps were developed to trap and reversibly deform giant unilamellar vesicles (GUVs) and in doing so manipulate membrane geometry and tension. We use these traps to show that filaments of the bacterial cytoskeletal protein FtsZ orientate themselves along the short axis of elongated GUVs and that membrane tension drives reversible FtsZ reorganization from filaments into rings, which facilitates membrane shape changes.

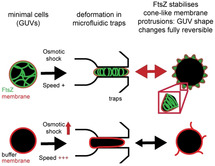

One hallmark of living entities is their ability to self‐organize into complex architectures, both on the level of cells and tissues, with molecular gradients controlling cell polarity, division, and the spatial dynamics of signaling.[^1^, ^2^] Interestingly, cell geometry itself can induce the required spatial symmetry breaks in signaling activity.^[3]^ In eukaryotes, cell shape is controlled by both plasma membrane properties and components of the membrane‐proximal cytoskeleton, most importantly the actin network.^[4]^ Recently, bottom‐up synthetic biology has been increasingly able to reconstitute these cellular phenomena in vitro, including spatially organized processes such as cell division.[^5^, ^6^]

Reconstituting cell division in vitro represents a desirable, albeit ambitious, goal towards the bottom‐up construction of an artificial cell.[^7^, ^8^, ^9^] Cell division involves the segregation of chromosomes and other intracellular components, concluding with cytokinesis, the physical splitting of the cell envelope. In bacteria, cytokinesis requires constriction and fission of the cell membrane as well as the peptidoglycan layer.^[10]^ Cell division in many bacteria involves the GTPase protein and tubulin homologue FtsZ.^[10]^ FtsZ polymerizes into a dynamic ring‐like structure at the division site (“Z‐ring”), where it is anchored to the membrane by adaptor proteins FtsA and ZipA.[^11^, ^12^, ^13^] The Z‐ring serves then as a platform to recruit further divisome proteins.^[7]^ The process of FtsZ assembly has been reconstituted in vitro at supported lipid bilayers.^[14]^ On these, FtsZ spontaneously assembles into dynamic ring structures, in which individual FtsZ filaments undergo treadmilling to drive chiral ring rotations.^[15]^ Reconstituting FtsZ in spherical aqueous droplets in oil showed dynamic FtsZ bundles, while studying FtsZ behavior inside rod‐shaped droplets was also attempted.^[16]^ FtsZ behavior in membrane‐bound compartments (polymersomes or giant unilamellar vesicles (GUVs)) has also been studied.[^8^, ^17^] However, bacteria are not spherical, and it is clear that cell shape has a great impact on the boundary conditions of these processes.

In the present study, we therefore introduce a single‐layer microfluidic chip based on previous work[^18^, ^19^] in which GUVs can be reversibly deformed into cigar‐like shapes by capturing them between narrowing PDMS posts. After several iterations (Figures S1–4), we found that the design shown in Figure 1 was optimal for GUV capture and deformation (Figure S1, design FS814): adjacent columns of traps (in 2 channels with 280 traps each) are vertically offset to maximize GUV capture, and post spacing is step‐wise reduced towards the chip outlet, such that differently‐sized GUVs are efficiently captured. Since we were interested in reconstituting elements of a synthetic cell division machinery in these elongated GUVs, we also designed the traps to mimic the “neck” of the division site (Figure 1 a). Each trap has multiple indentations for stable GUV trapping, and a “stopper” to prevent GUVs from escaping if they slip through the posts. Since the constriction features are small with a high aspect ratio (2×2×13 μm), we imaged both wafer using laser scanning based profilometry (Figure S5) and PMDS mold using scanning electron microscopy (SEM) for quality control (Figure 1 b). To test how encapsulated FtsZ filaments respond to compartment geometry, we made GUVs containing purified FtsZ‐YFP‐mts (henceforth referred to as FtsZ) in polymerizing conditions. The membrane targeting sequence (mts) included in the protein's C‐terminal domain circumvents the need for ZipA or FtsA by directly attaching FtsZ to the membrane, and YFP allows direct visualization of FtsZ polymerization.[^15^, ^20^, ^21^, ^22^] Importantly, this construct has also been shown to form functional Z‐rings in vivo.^[22]^ Using a syringe pump to apply negative pressure from the outlet to suck FtsZ‐containing GUVs (FtsZ‐GUVs) into the chip, we were able to capture and trap GUVs for minutes (flow rates 5–10 μL h^−1^, Figure 1 c). Some GUVs deformed from a spherical to an elongated shape (Figure 1 c, white arrow), but most trapped GUVs remained spherical. Using up to 50 times higher flow rates, we succeeded in “squeezing” GUVs fully into the trap funnels (Figure 1 d,e, bottom images). Interestingly, compared to GUVs only containing FtsZ buffer (control GUVs), FtsZ‐GUVs deformed strongly and at lower flow rates, but only if FtsZ was membrane‐bound and GTP was present (Figure 1 f, Figure S6a). This suggests that FtsZ filament attachment and treadmilling at membranes may affect spontaneous membrane curvature and hence extent of vesicle deformation obtained under forces. When we applied a mild osmotic deflation (14–23 % increase in buffer osmolarity), GUV deformation was greatly facilitated, even more so in the presence of FtsZ (Figure 1 f). GUVs assumed non‐spherical shapes likely more readily as osmotic deflation likely increased the membrane area‐to‐volume ratio, although we could not reliably quantify GUV volumes by confocal microscopy (see SI methods for further discussion). After the initial deformation flow rates of 5 μL h^−1^ were sufficient to keep GUVs stable in rod‐like shapes and the mean aspect ratio from ≈1 for spherical GUVs changed to 6.7±1.9 (Figure 1 g). We were thus able to create GUVs with a height of 13 μm and width of 7 μm (or 5 μm at the constriction sites), corresponding to the trap dimensions, and lengths up to ≈50 μm depending on initial GUV size (Figure S6b).

**Figure 1:**
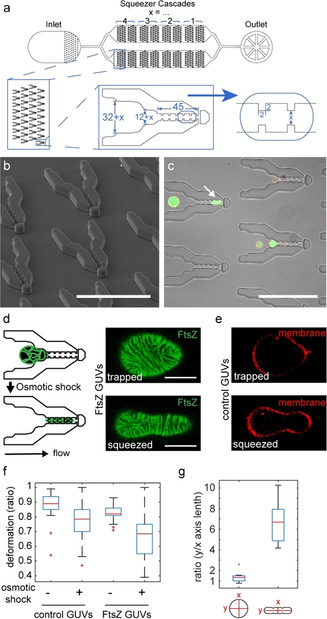
Design of microfluidic GUV traps. a) Schematic depiction of chip design, zooming in on the trap features. All dimensions in μm. b) SEM image of PDMS mold as a quality control step showing clean trap features. c) Brightfield and fluorescent microscopy composite image showing traps and trapped GUVs (containing FtsZ‐YFP‐mts, green and DOPE‐ATTO655 in the membrane, red). Scale bars for (b,c) are 100 μm. d) Schematic depiction and confocal images of trapped (top) and deformed (bottom) FtsZ‐GUVs (GUVs containing FtsZ‐YFP‐mts, green) and e) GUVs containing FtsZ buffer (control, DOPE‐ATTO655 in membrane, red). Scale bars are 10 μm. f) Maximum deformation of FtsZ‐ or control GUVs before and after osmotic deflation. g) GUV aspect ratios before and after squeezing. Box plots in (f,g) denote median in red, interquartile range as blue box, the 2.7σ (99.3 %) confidence interval as whiskers and outliers with a red cross. n(GUVs) >10 from >4 independent experiments.

In elongated FtsZ‐GUVs, FtsZ filaments aligned preferentially perpendicular to the GUVs’ long axis (Figure S7), particularly at GUVs’ necks (Figure 2 a,b). Releasing GUVs from the traps by reversing flow direction, they reassumed spherical shapes within a minute (Figure 2 c,d, S8a). Strikingly, this shape change was accompanied by FtsZ filament reorganization: elongated FtsZ filaments re‐polymerized into ring‐like structures (Figure 2 d, i‐ii). Although our image resolution is insufficient to discern filament organization, the dimensions make it likely that these structures correspond to the dynamic rings previously described (see Figure S9 for experiments under identical conditions to^[23]^). These ring‐like filaments formed in short cone‐like membrane protrusions at GUV surfaces, as soon as the mechanical membrane tension was decreased upon isosmotic GUV release from the traps (*σ*_mec_ (shape change)=−70±10 mN m^−1^; *n*(GUV)=10, Figure 2 e–f, Figure S8b; see SI for details on calculation of *σ*_mec_ from Figure S6 data). The change from filaments to rings was also apparent from a shorter mean filament length (Figure 2 g). Interestingly, we observed that osmotic deflation instead of FtsZ‐GUV trapping and elongation also induced FtsZ‐stabilized membrane protrusions (Figure S10a–c). Taken together, these results suggest that lowering elastic membrane tension, either by releasing external forces (GUV release from traps) or by osmotic deflation, both lead to FtsZ reorganization and FtsZ‐driven membrane shape changes. Importantly, higher solute concentrations alone, as expected for osmotically deflated GUVs, did not result in increased FtsZ‐stabilized membrane protrusions, but even at higher solute concentrations, FtsZ‐protrusions could still be reliably induced by osmotic deflation (Figure S10d).^[23]^ We also note that elastic membrane tension only affects membrane‐bound FtsZ, as for GUVs containing FtsZ without a membrane targeting sequence (FtsZ wt) FtsZ organization was unaffected by osmotic deflation (Figure S11a). In contrast to the short cone‐like protrusions observed for FtsZ‐GUVs, we observed that in control GUVs, the excess membrane area upon reobtaining spherical shapes was stored by the formation of longer and percolated membrane tubules, as previously described (Figure 2 h, white arrows). Similar tubules were also observed when relaxing mechanically‐tensioned GUVs containing FtsZ wt, confirming that FtsZ needs to be membrane‐bound to affect GUV membrane shapes (Figure S11b). In summary, our data suggest that FtsZ filaments reorganize on low‐tension membranes into ring‐like structures at membrane regions of higher curvature (“cones”), possibly driven by an intrinsic preference of membrane‐bound FtsZ for higher membrane curvature.

**Figure 2:**
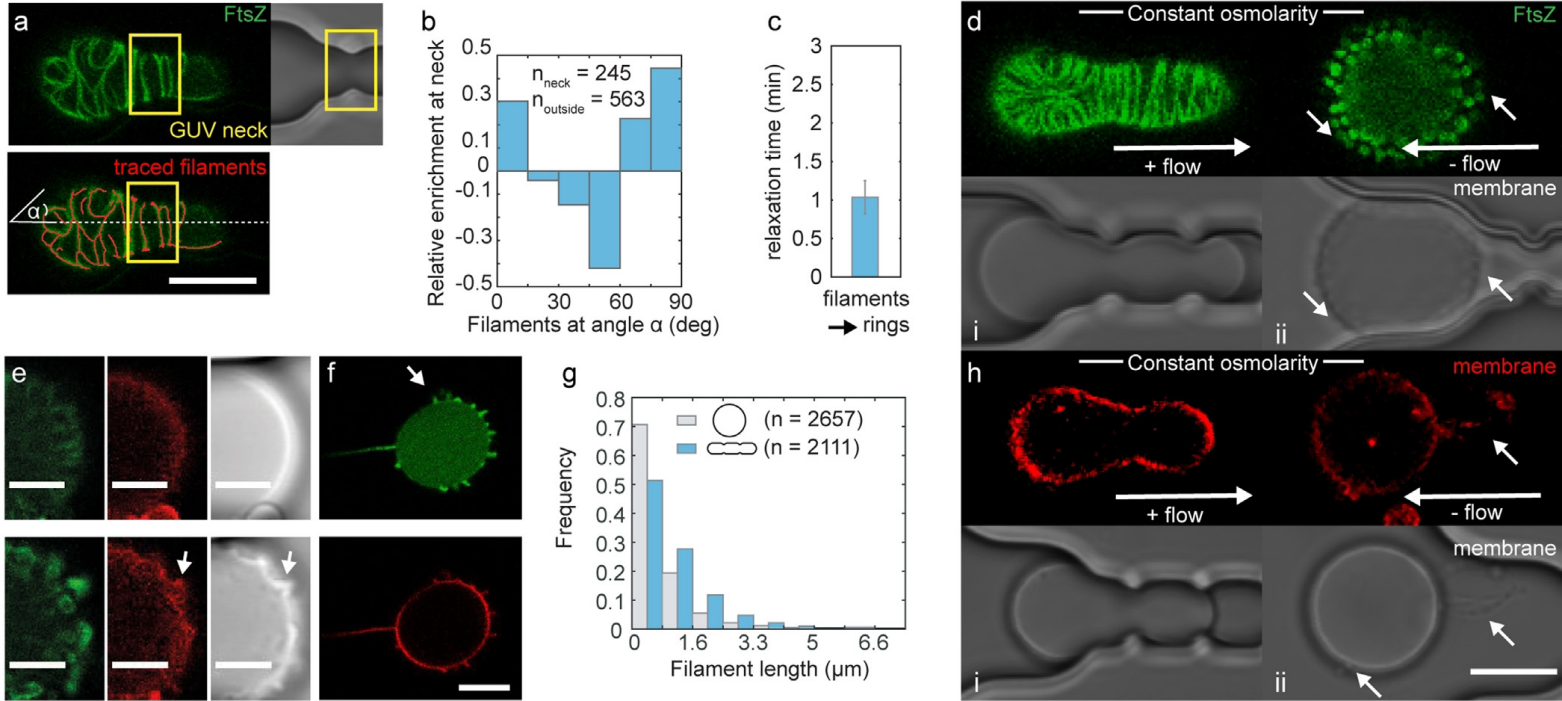
GUV deformation affects FtsZ filament alignment and filaments reorganize as constraints on GUV shape are released. a) Confocal images of trapped FtsZ‐GUVs (FtsZ in green, top), the same image overlaid with automatically traced filaments (red, bottom) and DIC image (right). Neck region marked with yellow box (manually defined from brightfield image). Scale bar is 10 μm. b) Relative enrichment of filaments at angles α with the GUV axis at GUV necks; n(filaments) >245. c) Mean relaxation times for FtsZ reorganization. Error bars are s.d. d,h) Confocal images of the equatorial plane of a trapped and reversibly elongated FtsZ‐GUV (d, i‐ii, top) or a control GUV (h, i‐ii, top, membrane labelled with DOPE‐ATTO655) and corresponding DIC images (d,h, bottom). The scale bar is 10 μm. e) Close‐up images of FtsZ reorganization, from left to right: confocal images of FtsZ‐GUVs (FtsZ, green; DOPE‐ATTO655, red) and corresponding DIC images. Elongated GUV (top) and the same GUV after isosmotic release from the trap, having reassumed a spherical shape (bottom). Scale bars are 5 μm. f) Membrane protrusions are filled with FtsZ. (FtsZ, green; DOPE‐ATTO655, red) Scale bar is 10 μm. g) Distribution of filament lengths in FtsZ‐GUVs. n is the number of filaments analyzed. For all experiments, n(GUVs) >12 from >4 independent experiments.

With membrane area being “stored” in membrane protrusions (cones), we tested whether these structures could act as membrane area reservoirs to facilitate deformation of GUVs into aspherical shapes, analogously to applying an osmotic shock before deforming GUVs. When we pushed FtsZ‐GUVs with membrane protrusions back into the traps after a first (osmotically facilitated) deformation cycle, we observed that deformation was now indeed fully reversible under isosmotic conditions, merely by changing flow direction (Figure 3 a, S12). Again, GUV shape changes were accompanied by FtsZ filament reorganization: in a few minutes, rings gave way to elongated filaments as protrusions were re‐incorporated into the membranes of elongated GUVs (Figure 3 a, i‐iii, S8, Movie 1). While almost all FtsZ‐GUVs repeatedly deformed, the majority of control GUVs did not (success rate of individual attempt ≈90 % for FtsZ‐GUVs versus <50 % for all controls, Figure 3 b). In addition, more deformation cycles could be achieved, on average, for FtsZ‐GUVs (up to 5, mean of 2.5 versus <2 for all controls, Figure S13a). Even when control GUVs could be successfully deformed, higher flow rates were required for shorter vesicle deformation compared to FtsZ‐GUVs (Figure 3 c, S13b). Taken together, our data suggest that membrane‐bound FtsZ filament reorganization facilitates GUV membrane shape changes. This facilitation required FtsZ to cycle between different polymerization states (rings and filaments) in an energy‐dependent (i.e. GTP‐dependent) process depending on overall membrane tension, allowing to reversibly store excess membrane area (Figure 3 d).

**Figure 3:**
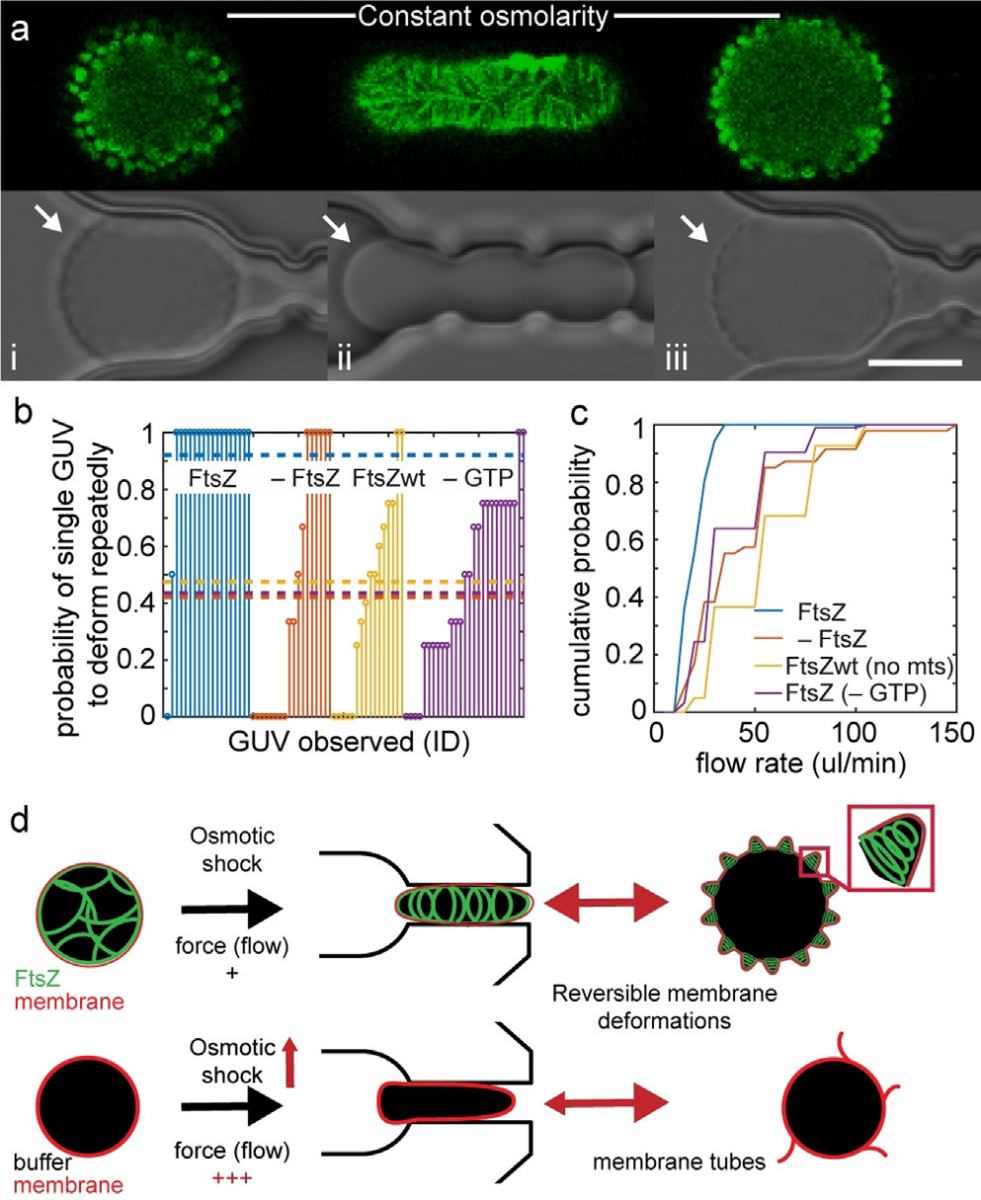
Reversible transitions between FtsZ filaments and rings allow reversible deformation of FtsZ‐GUVs. a) Confocal images of a trapped and elongated GUV containing FtsZ filaments (i‐iii, FtsZ shown in green, top) and corresponding DIC images (bottom). White arrows point to membrane topography changes. Scale bar is 10 μm. b) Probability that an individual GUV could be deformed five times for FtsZ‐GUVs or control GUVs without FtsZ (red), with FtsZ‐wt without membrane targeting sequence (‐mts, purple) and with FtsZ without GTP in the buffer (no dynamic FtsZ polymerization, yellow). The value for the mean deformation probability for each experimental condition is plotted as a horizontal line (color‐coded). c) Cumulative probability of flow rate required to deform FtsZ‐ or control GUVs (controls as in (b)). d) Schematic depiction of model: FtsZ structural reorganization from filaments to rings stores membrane reversibly in protrusions, facilitating transitions between GUV shapes. *n*(GUVs)=17–35 (controls) and 19 (FtsZ) from >10 independent experiments.

In summary, we have designed microfluidic traps for deforming GUVs with constriction sites mimicking the indentation of a division furrow and at mean aspect ratios close to that of rod‐like bacterial shapes. Fanalista et al. have also developed microfluidic traps to deform water‐in‐oil droplets to spheroid‐like shapes.^[24]^ In contrast to our observations, they reported FtsZ alignment along the long axis of deformed droplets.^[24]^ In their experiments, however, FtsZ is not membrane‐anchored which likely explains the discrepancy. In principle, our microfluidic chip can deform many GUVs in parallel. In practice, we studied single GUVs at a time to resolve the GUVs’ responses at maximal temporal resolution. However, the large number of traps was still advantageous to select specific GUVs (e.g. those encapsulating FtsZ) from a heterogeneous population.

Using these traps to deform FtsZ‐GUVs, we show that imposing a rod‐like geometry with a central membrane neck is sufficient to align FtsZ filaments along the short axis, preferentially at the neck location, mimicking FtsZ assembly into the Z‐ring at the division site in live bacteria. This is in agreement with previous results for elongated lipid‐bilayer coated containers or liposomes.[^14^, ^25^, ^26^] When rod‐shaped GUVs were released from the traps, GUVs relaxed back into a spherical shape with a lower surface/volume ratio. As expected, the concomitant excess of membrane area led to the formation of membrane tubules in absence of FtsZ. However, FtsZ‐GUVs showed highly‐curved membranes in form of cone‐like, FtsZ‐filled membrane protrusions as filaments reorganized into ring‐like structures, independently of GUV size. Low membrane tension has been shown to play an important role for FtsZ filaments to induce membrane shape changes:^[27]^ FtsZ reorganization similar to that observed by us has also been shown on deflated vesicles decorated with FtsZ on the outside.^[23]^

Interestingly, changes in membrane and filament organization were fully reversible: repeated GUV trapping and release cycled FtsZ‐GUVs between (1) a state of tense GUV membranes, with elongated FtsZ filaments and macroscopically smooth membrane surfaces, and (2) a state of relaxed GUV membranes, with GUV membranes showing protrusions stabilized by ring‐like FtsZ filaments (see Movie 2 and 3).^[23]^ In contrast, shape changes in control GUVs required increasingly higher forces and/or repeated osmotic deflation. Therefore, we conclude that FtsZ on its own is able to not only actively influence membrane shapes, but also facilitates externally‐induced changes by conserving and releasing excess membrane area (in an energy‐dependent process). Our results suggest that FtsZ organization into ring‐like filaments and FtsZ‐driven membrane deformation may only occur as membrane tension is decreased during the division process (e.g. by de novo membrane synthesis), and that this could contribute to controlling the timing of cell division machinery assembly and thus cytokinesis. Reorganized FtsZ filaments may then also constrict lipid membranes, generating forces by GTP‐fueled treadmilling.[^28^, ^29^]

Overall, we present this microfluidic device as a platform for studying the effect of compartment geometry and membrane tension on processes reconstituted in and on the membrane of GUV‐based minimal cells. In future experiments, these membrane tension changes could be measured quantitatively using tension‐sensitive dyes or by quantifying the hydrodynamic forces in our microfluidic devices.^[30]^ Beyond GUVs, our traps could also be used to deform vesicle‐like membranes derived from cells, such as spheroblasts^[31]^ and giant plasma membrane‐derived vesicles.[^32^, ^33^] Likewise, it will be interesting to study compartment geometry effects on other processes, such as MreB, the Min reaction‐diffusion system, or the cell‐polarity inducing Cdc42 system.[^34^, ^35^] With the increasing capacity to create artificial, minimal cells, for example, by encapsulation of cell‐free expression systems,^[36]^ a device such as the one presented herein will be useful for studying how compartment geometry affects the behavior of increasingly complex molecular networks.

## Conflict of interest

The authors declare no conflict of interest.

## Supporting information

As a service to our authors and readers, this journal provides supporting information supplied by the authors. Such materials are peer reviewed and may be re‐organized for online delivery, but are not copy‐edited or typeset. Technical support issues arising from supporting information (other than missing files) should be addressed to the authors.

Supplementary

Supplementary

Supplementary

Supplementary
