# Supplementary material for: FtsZ Reorganization Facilitates Deformation of Giant Vesicles in Microfluidic Traps
Source: Angew Chem Int Ed Engl. 2020 Sep 17;59(48):21372–6. doi: 10.1002/anie.202001928 (PMC7756778; doi:10.1002/anie.202001928)
Supplement: Supplementary file 1 — Supplementary [file ANIE-59-21372-s001.pdf]

Angewandte Chemie
Eine Zeitschrift der Gesellschaft Deutscher Chemiker

Supporting Information

FtsZ Reorganization Facilitates Deformation of Giant Vesicles in Microfluidic Traps**

Kristina A. Ganzinger[+], Adrián Merino-Salomón[+], Daniela A. García-Soriano, A. Nelson Butterfield, Thomas Litschel, Frank Siedler, and Petra Schwille**

anie_202001928_sm_miscellaneous_information.pdf
anie_202001928_sm_Movie1.mp4
anie_202001928_sm_Movie2.mp4
anie_202001928_sm_Movie3.mp4

Material and Methods

Chemicals

All reagents used in this work were from Sigma-Aldrich. Lipid compositions were prepared with DOPG (1,2-Dioleoyl-sn-glycero-3 phosphoglycerol, Avanti Polar Lipids, Inc.), EggPC (L-α-phosphatidylcholine (Egg, Chicken), Avanti Polar Lipids. Inc.), DOPC (1,2-Dioleoyl-sn-glycero-3-phosphocholine, Avanti Polar Lipids, Inc.) and DOPE-ATTO655 (ATTO-tec).

Microfluidic device fabrication

The microfluidic vesicle trap squeezer devices were fabricated from PDMS using rapid prototyping and standard soft lithography techniques. The geometry was inspired by the traps we used in a previous study[1], but here extended the trapping structure along the flow axis with a narrow squeezing channel containing evenly spaced indentations, and added a larger entrance funnel to increase GUV capture efficiency (Figure S1). We found it advantageous to design device channels with a high density of traps, which have progressively narrower funnels and squeezing channels towards the outlet.

Wafer fabrication and coating. Master moulds of 13µm height were produced on a 4-inch silicon wafer (University Wafer) using SU-8 3010 (Microchem corp.) according to the manufacturer's data sheet and developed in PGMEA (process parameters were optimized by direct laser writing (uPG101, Heidelberg Instruments, Germany) as described in Figure S2 to S4). Due to the small size of the PDMS features to be released from the mould, fluorophilic coating with Cytop was used routinely prior to the hard-baking step. In brief, 250 µL of a 1:10 dilution of Cytop CTL-809M in CTSOLV180 (Asahi Glass Co. Ltd., Japan) was dispensed on the SU-8 features and excess removed by spinning at 4000rpm for 1min. The coated wafer was then hard-baked for 30min at 180°C on a hot-plate and then allowed to cool down to room temperature slowly by turning off the heating. Quality control of the final SU-8 molds was done by laser profilometry (VKX1100, Keyence, Japan; Figure S5).

PDMS moulding. A 10:1 mixture of PDMS base and curing agent (Sylgart 184, Dow Corning) was homogenized and degased simultaneously for 2min using a Thinky Planetary Vacuum Mixer (ARV-310, Thinky Corp., Japan) and subsequently poured to about 4mm height onto the master in a petri-dish. After curing over-night in an oven at 75°C the PDMS was peeled off the wafer and cut to size. Fluid ports were then punched with a 3 mm and 0.75 mm diameter biopsy puncher (World-Precision-Instuments) for the reservoir and outlet, respectively. For quality control, images of PDMS chips were taken by Scanning Electron Microscopy (Mira3, Tescan) using sputter coated samples (1.5nm Pd/Pt, Cressington 208HR). In brief, samples were sputter coated with platinum/palladium on a high-resolution automatic sputter coater (Cressington 208HR) at 20 mA and 0.1mbar Ar for 3x 20s. Thickness of the applied coatings was measured with a build-in thickness controller to be 2.0 nm. Coated surfaces were viewed using a TESCAN MIRA3 FESEM operating at an accelerating voltage of 10 kV in SE mode. The microchannels of chips with well-resolved structures were then sealed by plasma bonding them onto glass cover slips (24 × 32 mm, thickness 1.5, VWR) using oxygen plasma (15 s at 0.3mbar, 50% power, model ZEPTO, Diener electronic, Germany) and baking them for 15–30 min at 75 °C on a hot-plate.

Preparation of FtsZ-containing GUVs

Giant unilamellar vesicles (GUVs) were prepared using a water-in-oil (w/o) emulsion transfer method.[2] The lipid-oil-suspension was made by first drying lipid films from lipids dissolved in chloroform (Uvasol): L-α-phosphatidylcholine (Egg, Chicken; EggPC) and 1,2-dioleoyl-sn-glycero-3-phospho-(1'-rac-glycerol (DOPG) in a ratio of 80:20 mol % (both Avanti Lipids), adding 0.02 or 0.07 mol % of 1,2-dioleoyl-sn-glycero-3-phosphoethanolamine (DOPE)-ATTO655 (ATTO-TEC) to label the lipid membrane. For this, the chloroform was evaporated from the lipid mixture by placed in a desiccator connected to a vacuum pump for >1h. The phospholipid-oil suspension was prepared by dissolving the lipid film in mineral oil (M5904, Sigma Aldrich) and sonicating the suspension for 30 min, reaching a homogeneous suspension with 0.5 mg/mL as final lipid concentration. GUVs were prepared by adding 15 µl of inner buffer into 500 µl phospholipid oil suspension pipetting carefully up and down to create a homogeneous emulsion. This emulsion was deposited over a lipid monolayer previously formed for >1h between 500 µl of phospholipid-oil suspension and 500 µl of the outer buffer in 2 ml Eppendorf tubes. The mixture was centrifuged for 10 min at 100 rcf to deposit the GUVs at the bottom of the tube. GUVs were collected and added into the inlet reservoir of the microfluidic chip or into microtitre plates for visualization.

Inner solution of FtsZ samples was prepared by diluting purified FtsZ-YFP-mts[3] to a final concentration of 2 µM in its buffer (25 mM Tris-HCl, 125 mM KCl, 6.25mM $MgCl_2$ pH 7.5) and adding 20% iodixanol (OptiPrep™, Sigma Aldrich) to increase the density of the encapsulated solution, in order to improve the vesicle yield obtained.[4] FtsZ samples were encapsulated in the polymeric form by adding 2 mM GTP and previously described GTP regeneration system (RS) to prolong the polymerised FtsZ lifetime[5]. Control FtsZ without GTP was prepared without the presence of the regeneration system. In all experiments, the outer and inner buffer solutions were the same but an additional 180 mM glucose was added to the outer solution to match the osmolarity of the inner solution (~480 mOsm/kg) (measured with Fiske Micro-Osmometer Model 210).

Samples containing FtsZ wt were prepared by diluting FtsZ wt to a final concentration of 2 µM including 0.8 µM FtsZ-wt-Alexa 488 as a fluorescent tracer. Both proteins were cordially provided by German Rivas' laboratory, purified and labelled as described.[5] (We note similar results were obtained using 1.65 µM, 1.4mM GTP and 2mM $MgCl_2$ as in reference [6](Figure S9). Experiments using GUVs with higher concentrated content were made by proportionally increasing all inner buffer components (including FtsZ, GTP regeneration system and iodixanol) by 20% or 50%. Glucose content of both outer solutions were also increased to match the osmolarities of the inner and outer buffers (620 mOsm/kg and 740 mOsm/kg for 20% and 50% respectively). Control GUVs without FtsZ were prepared using FtsZ buffer adding 160 mM glucose and 20% iodixanol to match the osmolarity of FtsZ-GUVs so the outer buffer was identical in all conditions.

We note that the yield of FtsZ-containing GUVs of desired sizes (10-20µm diameter) was variable even under identical experimental conditions, and that not all GUVs showed FtsZ filaments at the GUV membranes, likely due to variations in the final FtsZ concentration inside the vesicles. A high degree of variation in both vesicle content and yield is commonly observed for GUVs made by emulsion transfer. As we trapped many GUVs in our microfluidic device, we could focus our studies on those GUVs that initially showed visible FtsZ filaments when trapped and imaged by confocal microscopy.

Handling of microfluidic device and experimental setup

Microfluidic devices were passivated to prevent vesicle rupture upon contact with the walls by adding 20 μL of pluronic F-127 (Sigma Aldrich) at 10-50 mg/mL in phosphate buffered saline (PBS) in the inlet reservoir and centrifuging at 800 rcf for 10 min. The remaining pluronic was removed and cleaned with outer buffer. After passivation, the microfluidic devices were loaded with approximately 40 μl volume of vesicles in the inlet reservoir. A syringe joined to a pump system (neMESYS base 120 with neMESYS 290N, cetoni, Germany) and filled with 250 μl of ~60% ethanol was connected to the outlet of the device, avoiding any air in between the device and the syringe. Negative flow was applied at a rate of approximately 5-10 μl/h to draw the vesicle solution and reagents through the fluid channels during the experiments. After 10-20 min, a high number of vesicles were collected in the microfluidic traps. To deform the GUVs, we osmotically deflated them by replacing the buffer solution in the inlet reservoir with fresh buffer with a higher osmolarity. Some buffer exchanges with progressively higher osmolarity were sometimes necessary to induce vesicle deformation. Osmolarity was not increased more than 10-15% at a time to avoid vesicle rupture. Once the vesicles were partially deformed, the flow rate was progressively increased to introduce the GUVs completely into the trap, deforming them slowly. To move the vesicles out of the traps (and back in again, this time keeping osmolarity constant), the flow rate was usually changed within a range of ±15-20 μL/h for FtsZ experiments, although this flow rate range was increased depending on the conditions we tested. Our pump set up does not allow to calculate the forces exerted by the fluid flow on the vesicles inside the chip.

Experiments with FtsZ GUVs in the absence of trapping

FtsZ vesicles were deposited in a microtite plate for imaging (Greiner Bio-One, 364-well glass bottom SensoPlate™) previously passivated by 1 min of plasma cleaning (air plasma; model MiniFlecto®, Plasma Technology, Germany) and followed by a 30 min incubation with 10 mg/mL pluronic F-127 to avoid vesicle rupture. The plate was incubated tilted at ~45˚C for 10 min to favour vesicle accumulation in one of the borders improving thus their visualization. Vesicle deflation was then induced by replacing most of the buffer solution with fresh buffer with a higher osmolarity. For experiments with FtsZ GUVs, the osmotic difference varies from ~480 mOsm/kg in its initial buffer to ~560 mOsm/kg in the higher osmotic buffer, while for higher concentrated FtsZ vesicles, the osmolarity varies from ~620 to ~740 mOsm/kg in vesicles with 20% increased content and from ~740 to ~840 mOsm/kg for vesicles with 50% higher concentrated content. “Undetermined” category shown in Figure S9 includes vesicles with no protein in the membrane (depolymerized) and GUVs with no obvious filament or ring structures but showing protein binding to the membranes (likely meaning that the protein is polymerized in filaments smaller than the resolution of our confocal imaging (<1μm)).

Microscopy setup

All the experimental data were recorded with an LSM 780 confocal microscope (Carl Zeiss, Germany) equipped with a C-Apochromat, 40x/1.2 W objective. Fluorescence emission was detected by using laser excitation at 488 nm for YFP (FtsZ experiments) and Alexa 488 (FtsZ-wt

experiments), while 633 nm was used for Atto655. All the experiments were conducted at room temperature.

Image analysis

All image data was manually prepared for visualisation using ImageJ[7]. Image data was further analysed using ImageJ[7], either manually or using ImageJ plugins, or by MATLAB scripts as described in the following section.

Vesicle deformation (Figures 1f,g; 3b,c; S6; S12) was quantified using ImageJ by fitting an ellipse to the vesicle through the ATTO 655 laser channel. Aspect ratios in deformed or partially deformed vesicles were analysed by using the major and minor axis of the ellipses fitted in vesicles under different osmotic conditions and flow rates. Excel and MATLAB (2014/18b, The MathWorks; Statistics Toolbox) were used for plotting and descriptive statistics. For Figures 3b,c and S12a, the threshold for counting a GUV as successfully deformed was a deformation ratio of 0.7, which corresponds the mean value achieved for FtsZ-GUVs after an initial osmotic deflation.

Quantification of the volume change before and after osmotic shock was attempted but measurement scatter was very large, due to GUVs movement during z-stack acquisition, and as volume change was small this error was to large to allow us to discriminate volumes size before and after osmotic shock with confocal microscopy.

For Figure 2, filament angles were calculated using first the RidgeDetection plug-in from ImageJ (https://zenodo.org/badge/latestdoi/18649/thorstenwagner/ij-ridgedetection) that is based on the ridge / line detection algorithm by Steger.[8] The settings used were: line width '1.5', high Contrast '300', low contrast '100' and method for overlap resolution: 'slope'. The coordinates for the traced filaments were then exported to MATLAB via the ImageJ ROI tool for further automated analysis. We (1) calculated filament angles with respect to the GUV axis (Figure S7) and assigned whether they were positioned in the 'neck' region of the elongated GUV or not (Figure 2b; this region was manually defined around trap feature using the bright field image) and (2) analysed filament lengths and plotted the distribution of filament lengths for trapped (elongated) and released (spherical) vesicles (Figure 2g). Calculation of relaxation times (Figure 2c; S8a) was done manually by counting frames between trapping/releasing GUVs and complete resolution of FtsZ filaments or rings/protrusions.

Filament and ring detection in experiments with non-trapped vesicles (Figure S9) was done by collecting Z-stacks images from different positions selected in a tile image of the whole sample well. Z-stack spacing 2 μm was selected to optimize the vesicle visualization. The same procedure is repeated after the addition of the higher osmolarity fresh buffer to deflate the vesicles. Addition of new buffer involves a vesicle movement that makes difficult to track single vesicle state before and after the osmotic change. Filament and Ring detection was done manually using ImageJ. Further data analysis was done using MATLAB and Excel.

Estimate of mechanical membrane tension upon iso-osmotic GUV release from geometric confinement (microfluidic traps) from microscopy data

To infer the change in membrane tension that a GUV experiences as it is iso-osmotically released from the traps, we determine the GUV shape transformation upon trapping from our microscopy data and hence extrapolate the new GUV's surface area for elongated GUVs. In the absence of external forces or constraints (*i.e.* after release from the traps), a GUV membrane aims to attain

a new optimal area A_{opt} for its now spherical shape, which corresponds to the optimal packing of its molecules. The membrane thus experiences a mechanical tension, σ_{mec}, when its original area A deviates from this new optimal area A_{opt}.This mechanical tension can be expressed as[9]

$\sigma_{mec}(A) = K_A \frac{A-A_{opt}}{A_{opt}}$ with K_A being on the order of $200\frac{\mathrm{mN}}{\mathrm{m}}$ for lipid bilayers.[10]

Measuring the length and height of the deformed vesicles (Figure S6) and assuming a cylindrical shape, we obtain A_C and V_C of the cylinder. Then we can calculate the surface area A_S upon reassuming a spherical shape (at constant volume V_C=V_S). With $\frac{A-A_{opt}}{A_{opt}} = \frac{A_S-A_C}{A_C}$:

$$\sigma_{mec}(A_S) = -70 \pm 10\frac{\mathrm{mN}}{\mathrm{m}};\ n(GUV) = 10$$

References

[1] Q. Wang, M. Taschner, K. A. Ganzinger, C. Kelley, A. Villasenor, M. Heymann, P. Schwille, E. Lorentzen, N. Mizuno, *Nat. Commun.* **2018**, *9*, 4684.

[2] L. L. Pontani, J. van der Gucht, G. Salbreux, J. Heuvingh, J. F. Joanny, C. Sykes, *Biophys J* **2009**, *96*, 192–198.

[3] M. Osawa, D. E. Anderson, H. P. Erickson, *Science (80-.).* **2008**, *320*, 792–794.

[4] T. Litschel, B. Ramm, R. Maas, M. Heymann, P. Schwille, *Angew. Chemie Int. Ed.* **2018**, *57*, 16286–16290.

[5] J. M. González, M. Jiménez, M. Vélez, J. Mingorance, J. M. Andreu, M. Vicente, G. Rivas, *J. Biol. Chem.* **2003**, DOI 10.1074/jbc.M305230200.

[6] D. A. Ramirez-Diaz, A. Merino-Salomon, F. Meyer, M. Heymann, G. Rivas, M. Bramkamp, P. Schwille, *bioRxiv* **2020**, 587790.

[7] C. T. Rueden, J. Schindelin, M. C. Hiner, B. E. DeZonia, A. E. Walter, E. T. Arena, K. W. Eliceiri, *BMC Bioinformatics* **2017**, *18*, 529.

[8] C. Steger, *IEEE Trans. Pattern Anal. Mach. Intell.* **1998**, *20*, 113–125.

[9] R. Lipowsky, *Adv. Colloid Interface Sci.* **2014**, *208*, 14–24.

[10] E. Evans, D. Needham, *J. Phys. Chem.* **1987**, *91*, 4219–4228.

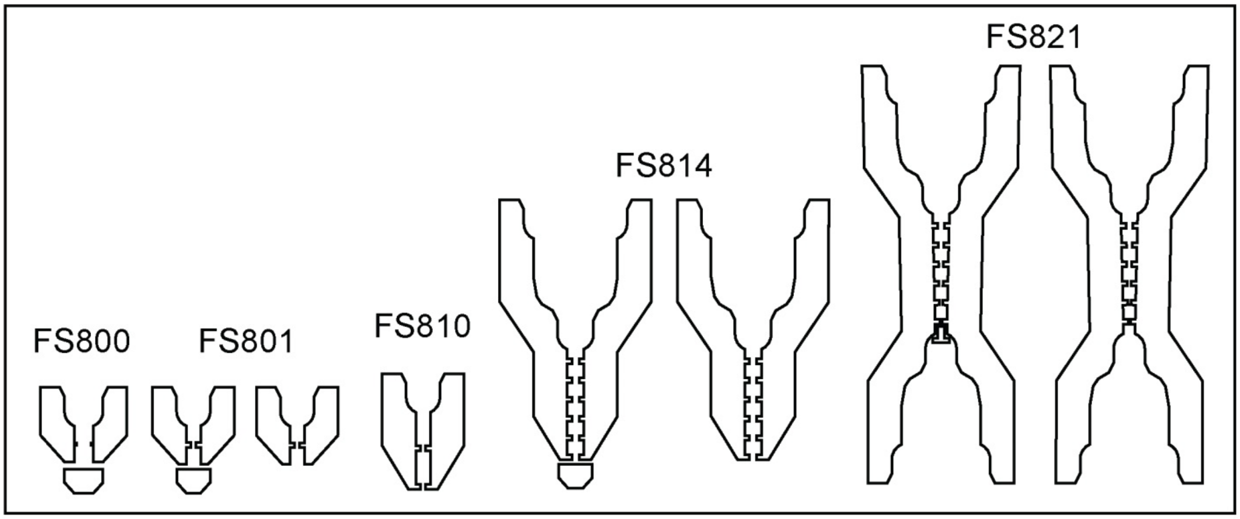

Figure S1. Evolution of microfluidic designs. To trap and squeeze GUVs in a microfluidic environment funnel like structures with various features were designed and tested. Structures are named by Experiment-ID. The central idea in the beginning was to capture GUVs in a funnel and then, by carefully rising the flow, to squeeze them past the subsequent neck. Indentations were intended to mimic the naturally occurring constriction site that is a part of cell division. Finally, the addition of stoppers was tested to prevent GUVs from escaping the traps too early.

In FS800 indentations were too small to be reproduced sufficiently precise and they did not induce a clear indentation of membranes of trapped GUVs. With FS801, a stopper was found to be necessary but the overall design showed already a reliable performance, and therefore all necessary exposure optimizations were done with this design. For some bigger GUVs, however, the relatively short neck section was a problem. Our preferred design was finally found with FS814, which was used in combination with a stopper for all experiments reported in this work. The prototype FS821 with a double funnel to facilitate back and forth shifting of GUVs and a slight narrowing of the central channel was not found to show any advantages.

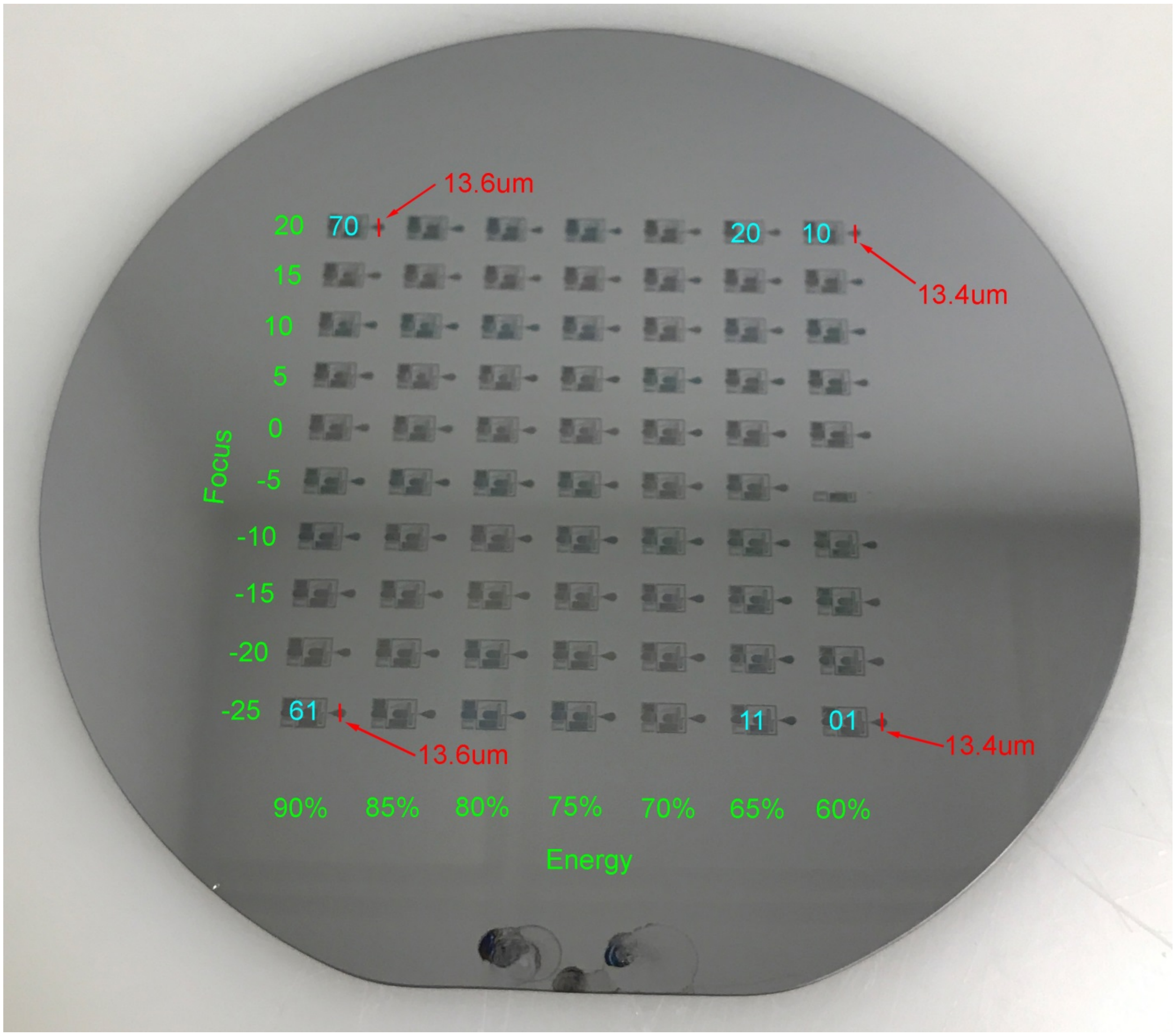

Figure S2. Optimization of Exposure Parameters. To find the best set of parameters for rapid prototyping via direct laser writing, a 7x10 array of test structures comprising the most delicate structural features was exposed on a 4 inch silicon wafer. Parameters varied were focus setting (y-direction; arbitrary units) and energy attenuation (x-direction; % of 10mW). For unique identification positions in the array are numbered from bottom to top and right to left as indicated by cyan numbers. Results of height measurements by laser profilometry are indicated in red.

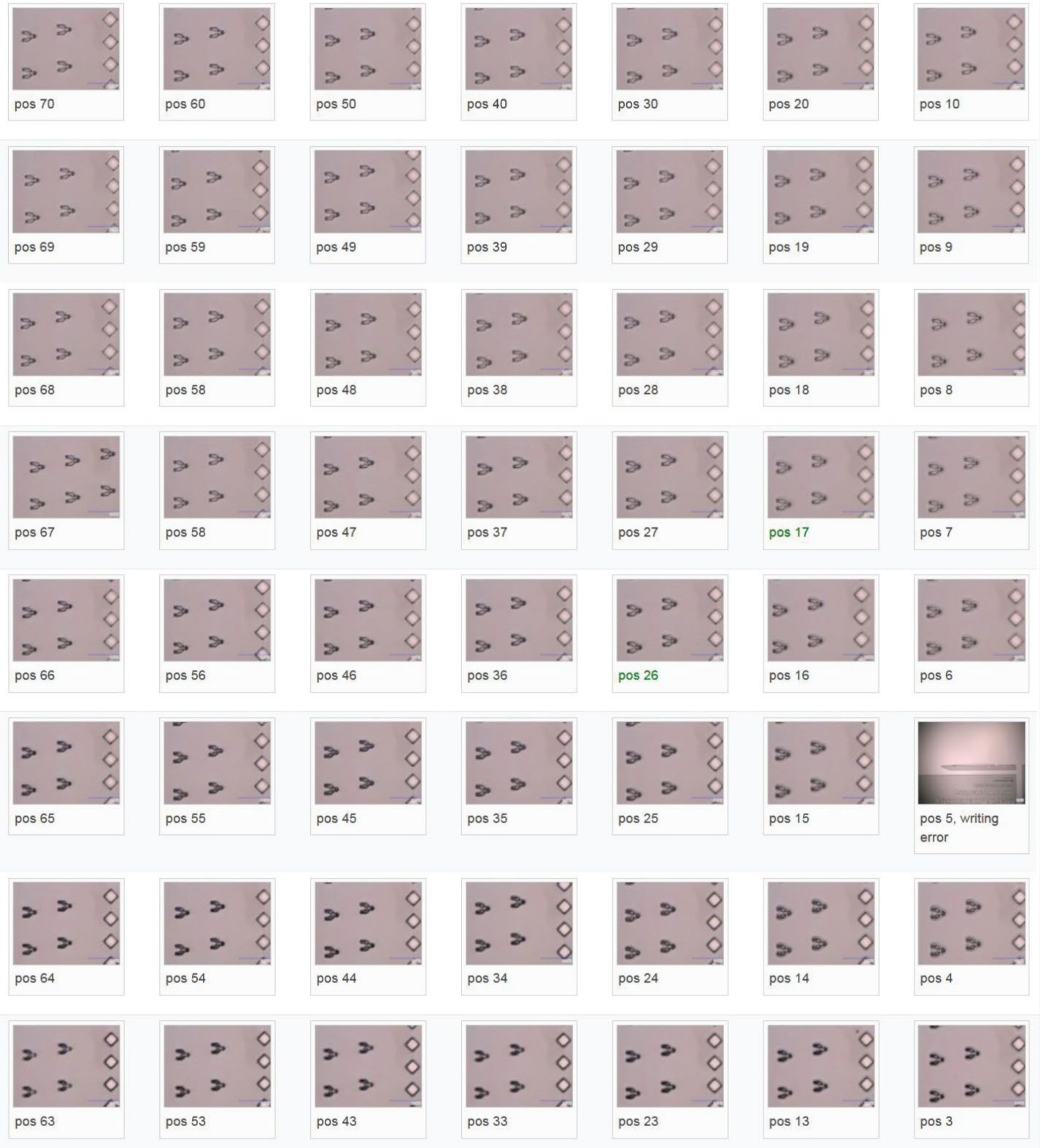

Figure S3. Light-microscopic inspection of the test array from Fig.S2. For all positions, images were taken from the same part of the test structure at constant microscopic settings. This overview is best suited to narrow down the parameter space to useful values. Close-ups indicating the subsequent decision making process are shown in Fig.S4

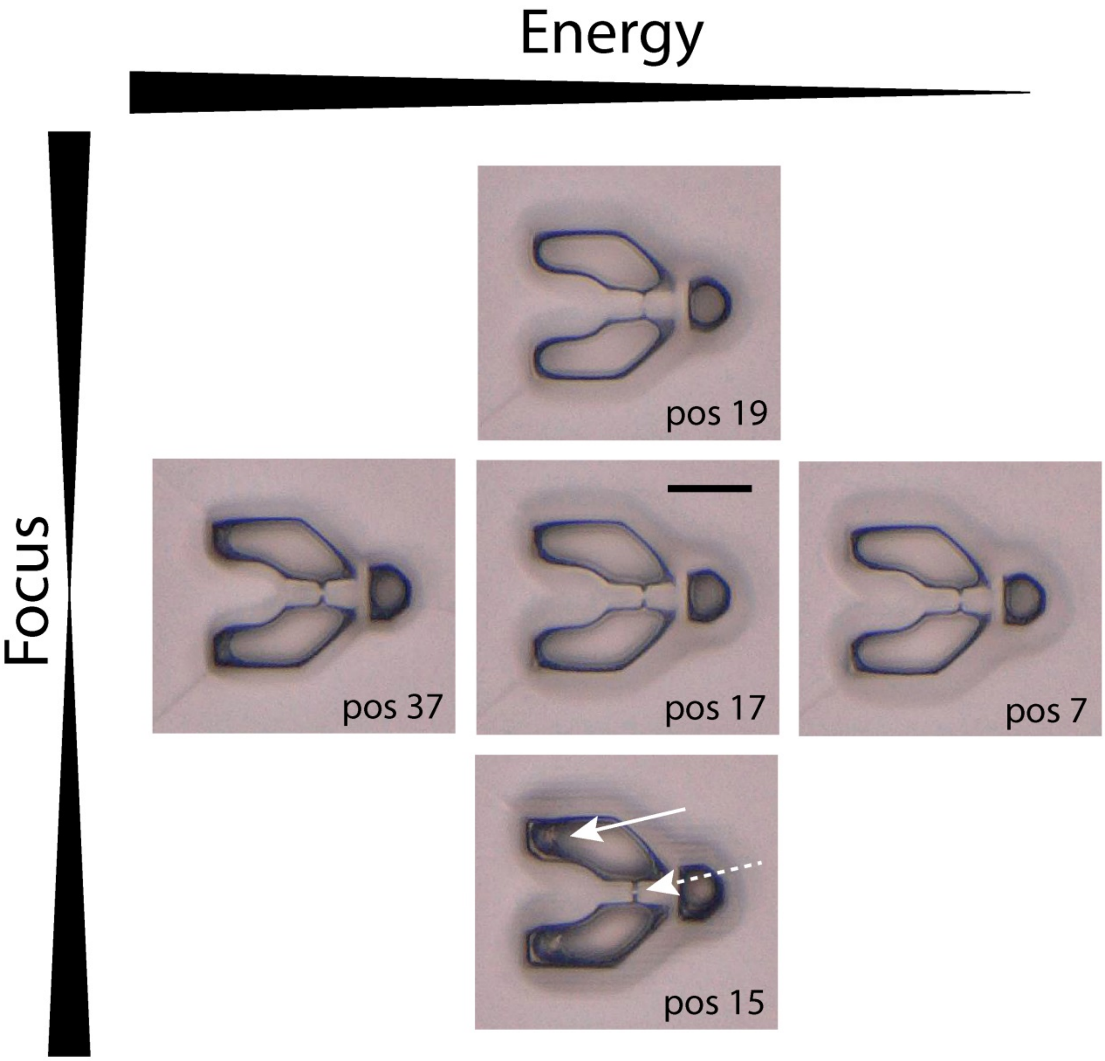

Figure S4. Optical inspection of the test structures from the array (Fig. S3) by light microscopy. In the course of the experiment, exposure energy and focus correction were varied as indicated on the top and left. Position numbers correspond to the ones given in the overview (Fig.S3). Areas important for quality judgement are marked by white arrows. Changing focus correction from negative values to zero (pos15 -> pos17) reduces unwanted reflections on the wafer surface (solid arrow) at the expense of sharpness (dashed arrow). Shifting focus correction even further to the positive (pos19) shows clearly a blurring of the structure.

A similar effect can be observed for the energy setting: Too high energy settings lead to reflections at the bottom and therefore cure resist in unwanted regions (pos37), whereas too low energy settings produce too soft and therefore undefined structures (pos7). In both directions, the best compromise appeared to be in pos17. Starting from these settings, the best parameter set for production could be estimated and tested (resist: SU8-3010, 13um height; laser writer: Heidelberg Instruments uPG101, 2mm writing head, energy 67% of 10mW, defoc +2). Scale bar 20µm.

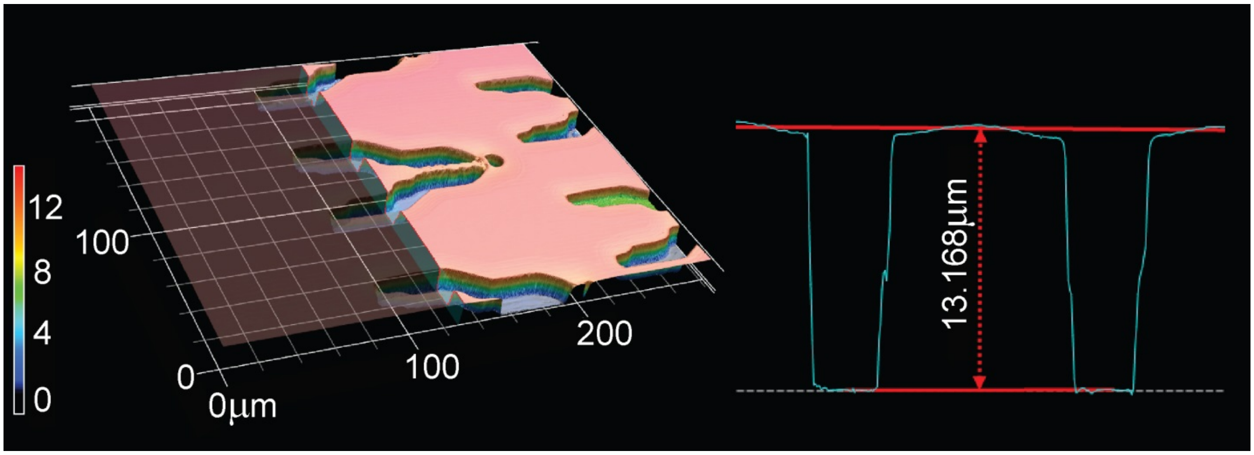

Figure S5. Quality control of final SU8 mold. The exact 3D structure of the final mold (FS814) was determined using laser profilometry (Keyence VKX1100). In the landscape representation (left), the smooth surface on the top and the intended steepness of the shoulders is visible. Height measurements on the 3D dataset can be done at any location (right).

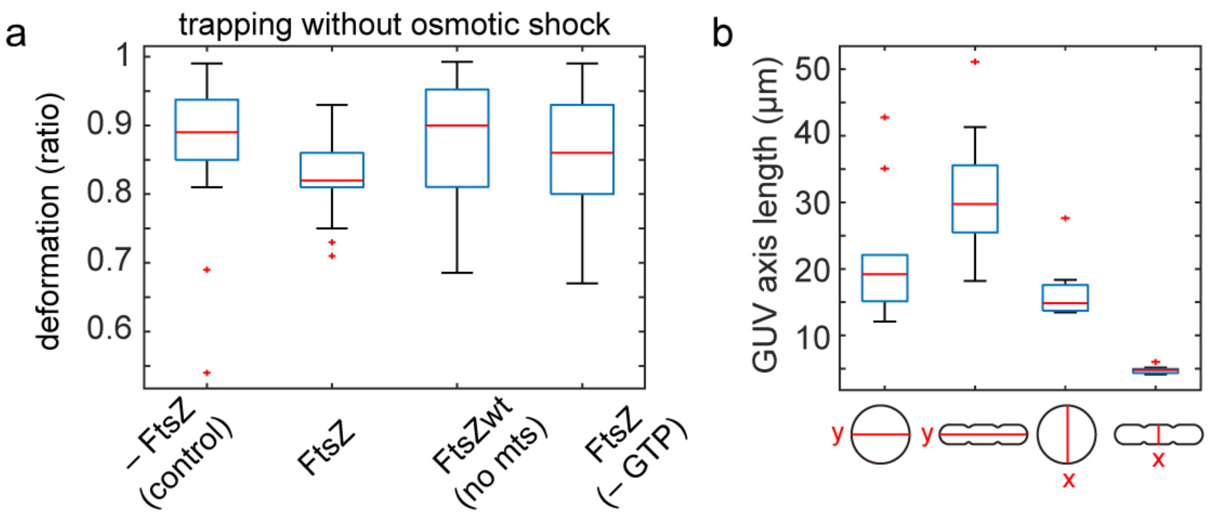

Figure S6 Maximum deformation of FtsZ- or control GUVs before osmotic deflation and determination of GUV aspect ratios after trapping and osmotic shock. a) Deformation of FtsZ- or control GUVs before osmotic deflation. Additional controls for Fig. 1f; data for GUVs with FtsZ-mts and without FtsZ is plotted again for comparison and identical to that of Fig. 1f. Additional controls are FtsZ-wt without membrane targeting sequence (mts) and with FtsZ without GTP in the buffer (no dynamic FtsZ polymerisation). b) The aspect ratio of trapped GUVs was manually determined in ImageJ at GUV mid plane; x and y axis length (input for the y/x ratio plotted in Fig. 1F). Box plot denotes median in red, interquartile range as blue box, the 2.7σ (99.3%) confidence interval as whiskers and outliers as red dots. n(GUVs) = 10.

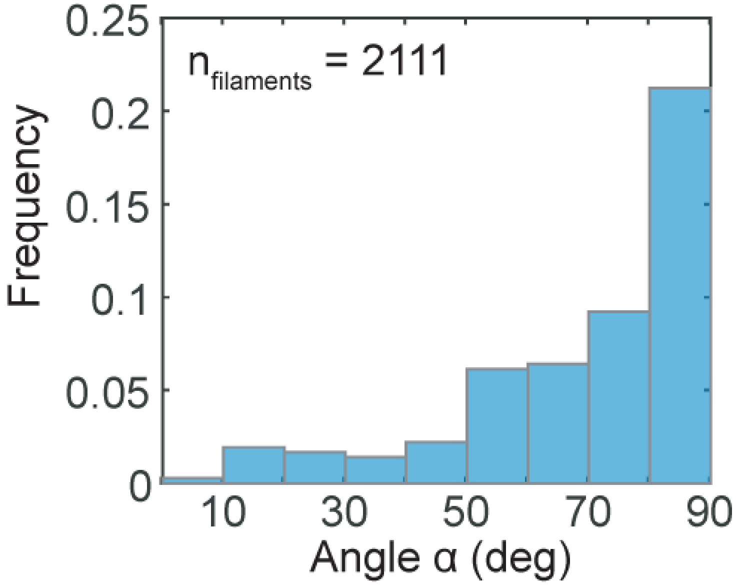

Figure S7 Orthogonal FtsZ filament alignment to the long axis in elongated GUVs. Elongation and indentation of GUVs containing FtsZ filaments leads to filament alignment orthogonal to the long axis at the GUV neck. Distribution of filament angles α for all filaments observed in maximum intensity projections of elongated GUVs. Angles were calculated as described in the methods section.

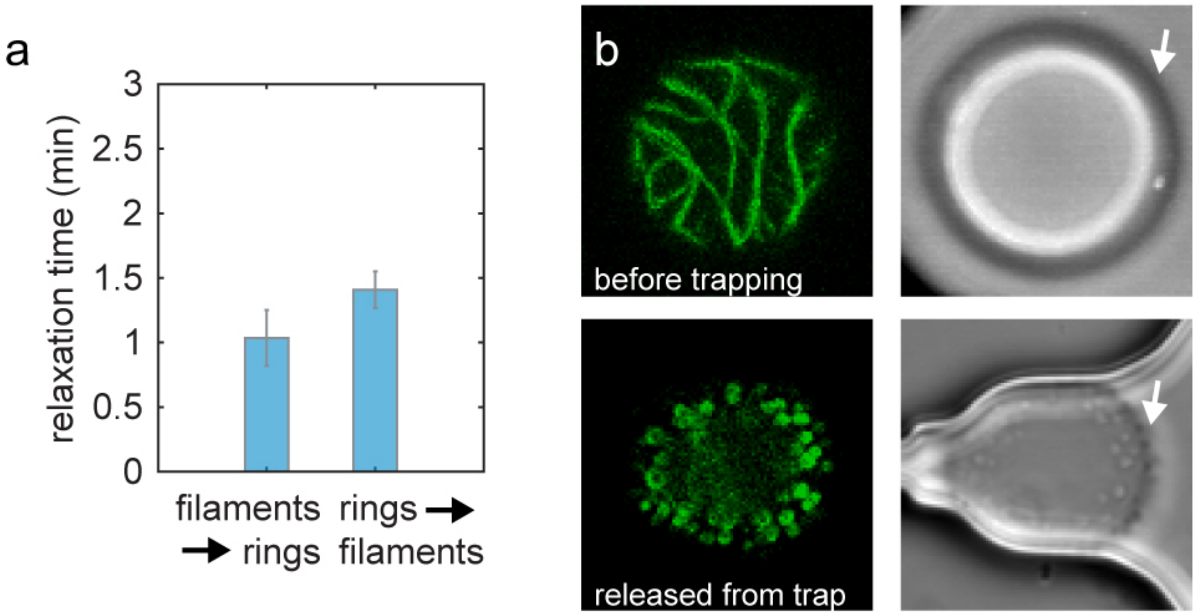

Figure S8 Upon membrane deflation (release from geometric confinement to spherical shape), FtsZ reorganises into dynamic rings that result in membrane protrusions. a) relaxation times for filament to ring transitions as observed by confocal microscopy of GUVs that were pushed out (filament -> rings) or into (rings -> filaments) traps. b) Confocal and corresponding bright field microscopy images of GUVs containing membrane-bound FtsZ filaments (green); example for elongated filaments observed in an untrapped GUV and ring-like filaments observed in a GUV after release from the trap, having assumed a spherical shape again. Note that the membrane shows protrusions after FtsZ filaments have rearranged into dynamic rings (bottom DIC image, white arrow) that are absent in the presence of long filaments (top DIC image, white arrow). Osmolarity was not changed in this experiment.

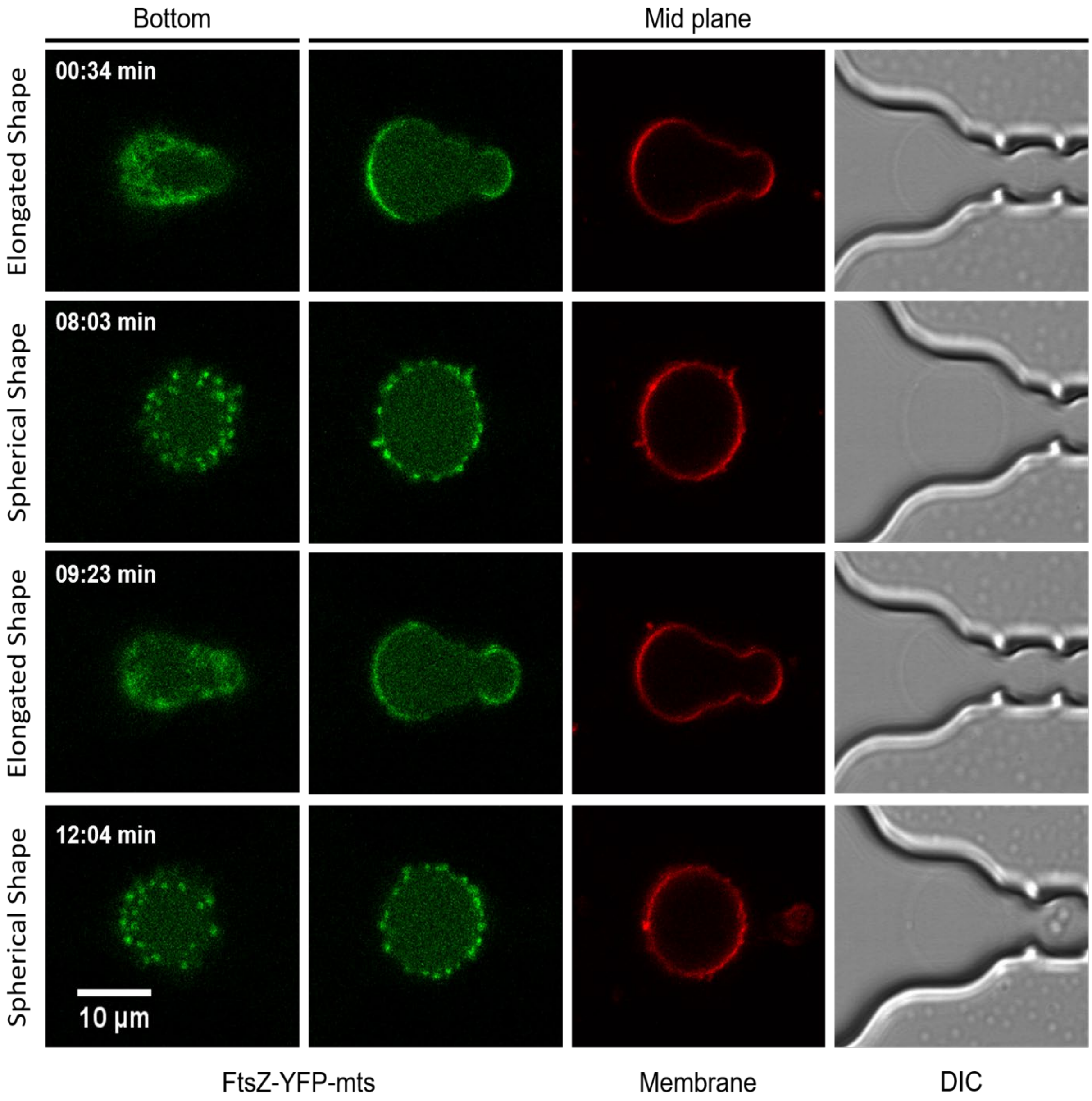

Figure S9 Different buffer conditions does not affect the behaviour of FtsZ. Slightly different buffer and protein conditions previously used in reference [6] were tested, showing that FtsZ is able to form membrane protrusions and filaments under this condition (1.65 μM FtsZ-YFP-mts and 1.4 mM GTP in buffer 125 mM KCl, 25 mM Tris-HCl, 2 mM $MgCl_2$), in agreement with results in our working buffer. Confocal images of a trapped and elongated FtsZ GUV into our microfluidic device. Images of FtsZ-YFP-mts (Green) at the bottom (left), and equatorial plane of the lipid vesicle.

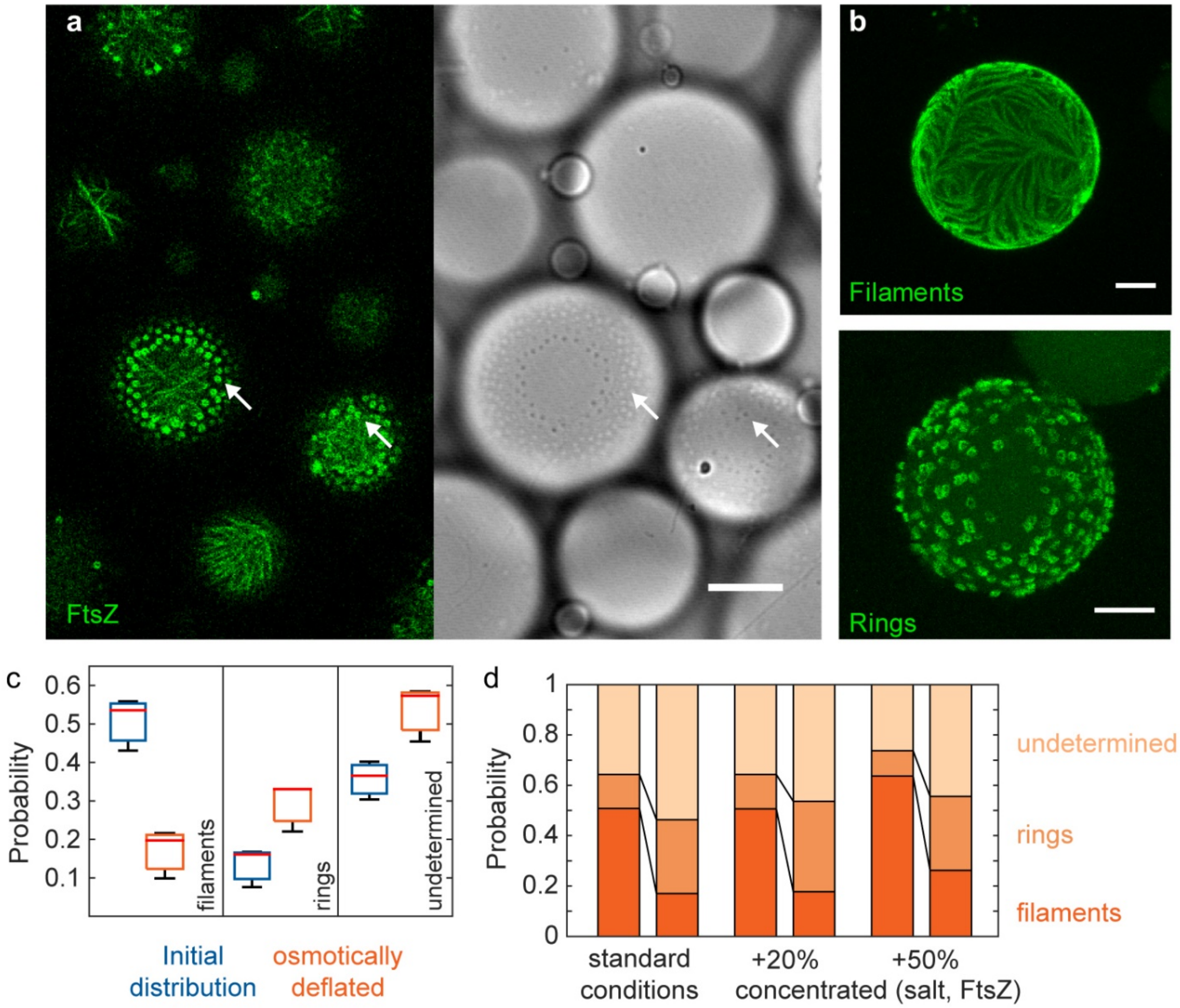

Figure S10 Osmotic deflation alone favours FtsZ reorganization into dynamic rings. a) confocal and corresponding bright field microscopy images of non-trapped GUVs in a microtitre plate after an osmotic shock; some GUVs with FtsZ show membrane protrusions (white arrows, bright field image) with ring-like FtsZ filaments (white arrows, green). Scale bar is 10 µm. b) Maximum intensity projection of a GUV with elongated FtsZ filaments (top) and rings (bottom). Scale bars are 5 µm. c) Quantification of GUV populations (FtsZ) showing FtsZ organisation (filaments, rings, undetermined) before or after osmotic deflation. These results show that a mild deflation of FtsZ GUVs is enough to lead FtsZ filament reorganisation into dynamic rings when GUV membranes are not concomitantly tensioned by the microfluidic traps. Box plots denote median in red, interquartile range as blue box, the 2.7σ (99.3%) confidence interval as whiskers and outliers as red dots. d) Quantification of GUV populations showing FtsZ filament organisation (filaments, rings, undetermined) before or after osmotic deflation for GUVs made with our standard buffer conditions and GUVs made with buffers of higher concentrations of buffering components and FtsZ. Note that higher salts/FtsZ concentrations alone do not favour ring formation unless GUVs are osmotically deflated. Data are from >3 independent experiments and >100 GUVs per condition.

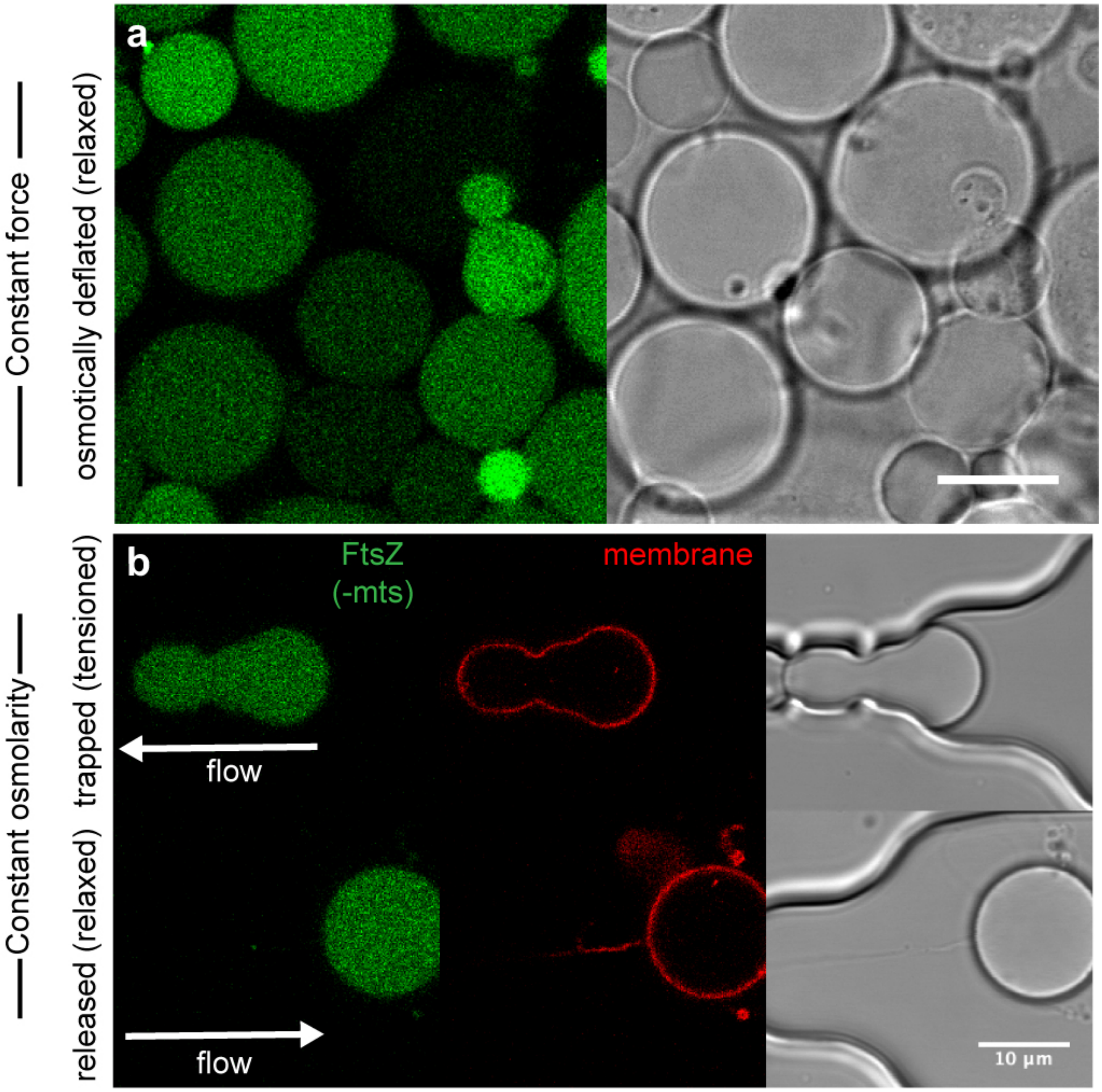

Figure S11 Membrane attachment of FtsZ is required to shape GUV membranes (into cone-like membrane spikes) upon relaxation from mechanically tensioned shapes or upon osmotic deflation. a) confocal and corresponding bright field microscopy images of non-trapped GUVs in a microtitre plate after an osmotic shock, GUVs with FtsZ-wt without membrane targeting sequence (mts) show no FtsZ-stabilised membrane protrusions after osmotic deflation b) Confocal images of the equatorial plane of an elongated (trapped) GUV (top row) and the same GUV after isosmotic release from the trap (bottom row), having reassumed a spherical shape. GUV contains FtsZ-wt without membrane targeting sequence (left, FtsZ wt-Alexa 488, green; middle, membrane labelled with DOPE-ATTO655; right, corresponding DIC images). Scale bar are 10 µm. Data is representative of GUVs from 12 independent experiments.

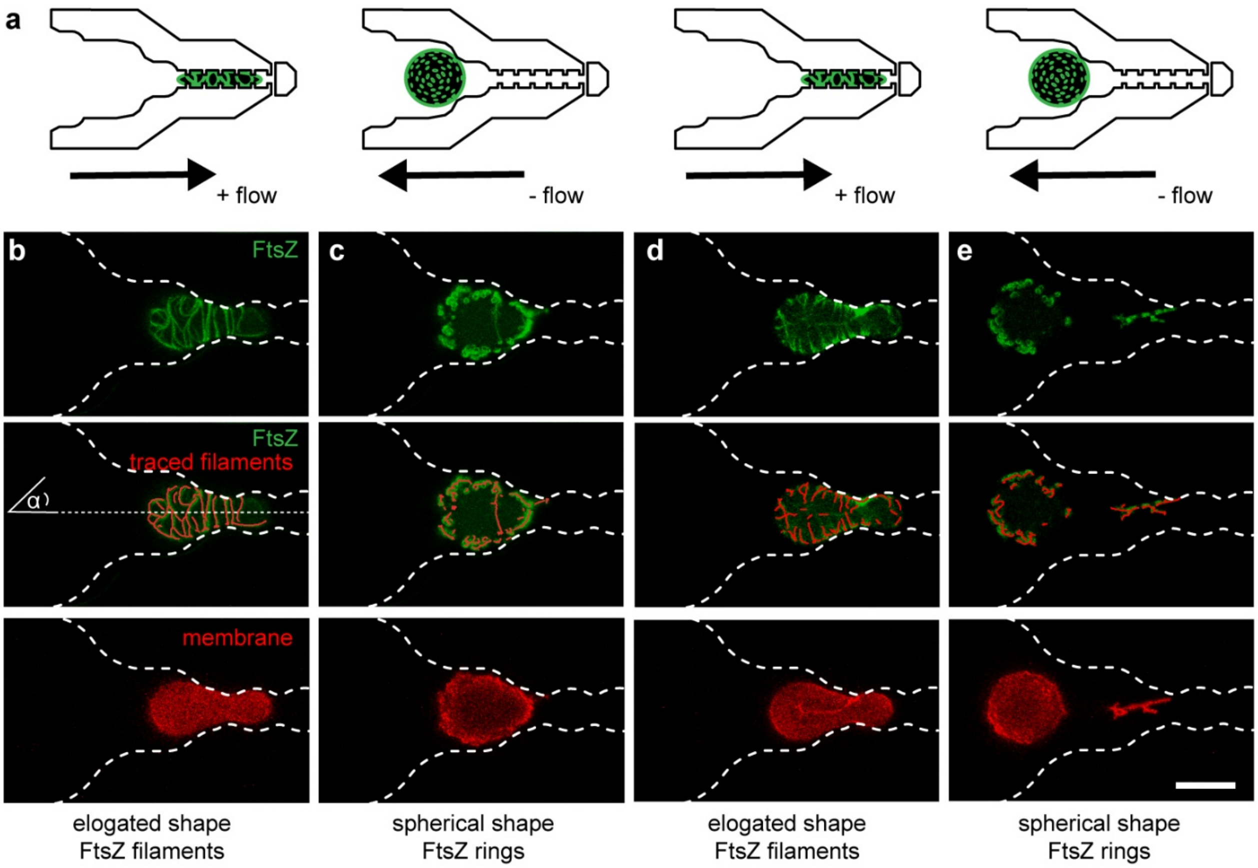

Figure S12 Reversible elongation and relaxation of FtsZ GUVs leads to transitions between FtsZ rings and long filaments. a) Schematic depiction of experiment. FtsZ forms elongated filaments in rod-shaped (tense) vesicles while FtsZ rings are formed when the vesicle is relaxed back to its spherical shape; b) confocal images of the equatorial plane of a trapped and elongated GUV showing FtsZ filaments (top), traced filaments (middle) and GUV membrane (bottom); FtsZ is shown in green. Trap outline is marked (white dotted line). c,d,e) transition of the same GUV between spherical c) and e) and elongated d) shapes purely by manipulation of flow rates and direction. Flow range was -15 to +10 ul/h and osmolarity was kept constant. Data is representative of GUVs from 12 independent experiments. The scale bar is 10 μm

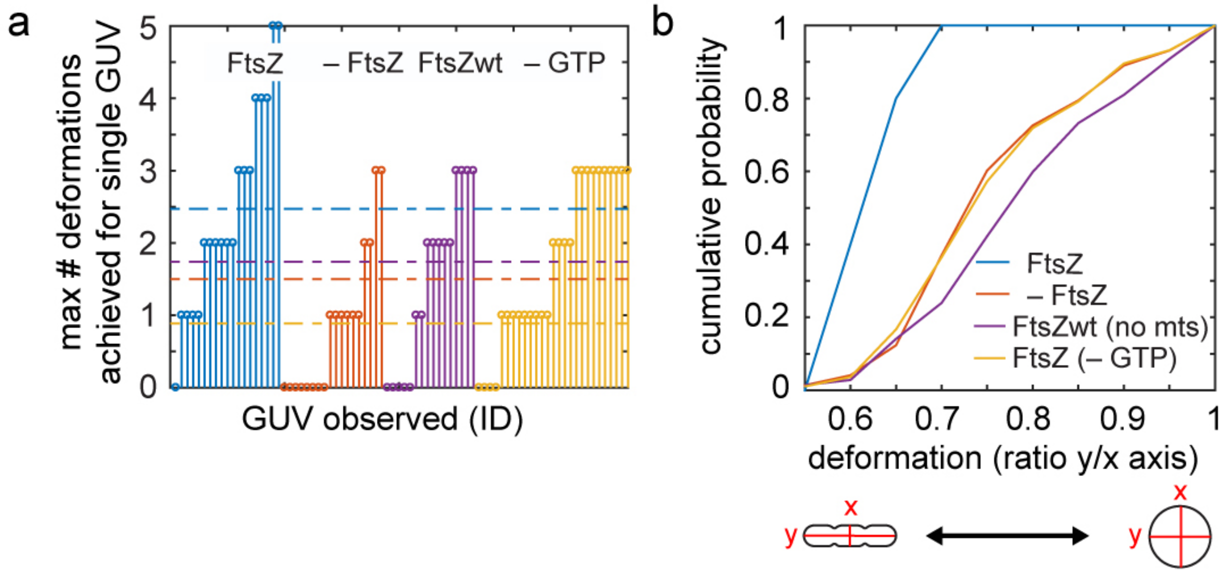

Figure S13 GUVs with a membrane-bound, dynamically polymerising FtsZ cytoskeleton can be deformed more often and more strongly under the same conditions (osmolarities, flow rates). a) Maximum number of deformations achieved for individual GUVs by repeated trapping for GUVs containing FtsZ or control GUVs without FtsZ (red), with FtsZ-wt without membrane targeting sequence (- mts, purple) and with FtsZ without GTP in the buffer (no dynamic FtsZ polymerisation, yellow). Stem plot shows # of deformations achieved for a single GUV; the value for the mean # deformations for each experimental condition is plotted as a horizontal line (colour coded). b) Cumulative probability of GUV deformation ratio achieved by trapping FtsZ or control GUVs without FtsZ (red), with FtsZ-wt without membrane targeting sequence (- mts, purple) and with FtsZ without GTP in the buffer (no dynamic FtsZ polymerisation, yellow). All osmolarities >480 mOsm/kg (internal osmolarity).

Movie legends

Movie 1 FtsZ-GUV can be reversibly elongated and relaxed into spherical shape. The movie shows the filament/ring transitions and formation of membrane protrusions generated by FtsZ rings. Osmolarity of the surrounding buffer remains unchanged. Green and red channel correspond to FtsZ-YFP-mts and lipid membrane (DOPE-ATTO 655), respectively. For the video, several confocal movies (played back at 80x) were stitched together. Therefore, time intervals are not constant throughout the entire videos as we do not know the precise time interval between individual videos.

Movie 2 FtsZ-GUV can be deformed into rod- or cigar- shapes using the microfluidic devices. The movie shows reversible squeezing and elongation of a FtsZ-GUV by controlling the flow rate in the device microchannels. Osmolarity of the surrounding buffer remains unchanged. Green channel corresponds to FtsZ-YFP-mts while red channel corresponds to lipid membrane (DOPE-ATTO 655). For the video, several confocal movies (played back at 70x) were stitched together. Therefore, time intervals are not constant throughout the entire videos as we do not know the precise time interval between individual videos.

Movie 3 Continuous time-lapse of reversible FtsZ-GUV deformations in microfluidic devices. The flow rate used in this video varies from -20 µl/h to +5 µl/h to push in and out the vesicle. Reversible transition between FtsZ filaments and rings can be observed. Green and red channel correspond to FtsZ-YFP-mts and lipid membrane (DOPE-ATTO 655), respectively. Two first digits of the time counter represent minutes while the last two correspond to seconds (the movie is played back at 150x).
